# A bi-objective integer programming model for partly-restricted flight departure scheduling

**DOI:** 10.1371/journal.pone.0196146

**Published:** 2018-05-01

**Authors:** Han Zhong, Wei Guan, Wenyi Zhang, Shixiong Jiang, Lingling Fan

**Affiliations:** MOE Key Laboratory for Urban Transportation Complex Systems Theory and Technology, Beijing Jiaotong University, Beijing, China; Southwest University, CHINA

## Abstract

The normal studies on air traffic departure scheduling problem (DSP) mainly deal with an independent airport in which the departure traffic is not affected by surrounded airports, which, however, is not a consistent case. In reality, there still exist cases where several commercial airports are closely located and one of them possesses a higher priority. During the peak hours, the departure activities of the lower-priority airports are usually required to give way to those of higher-priority airport. These giving-way requirements can inflict a set of changes on the modeling of departure scheduling problem with respect to the lower-priority airports. To the best of our knowledge, studies on DSP under this condition are scarce. Accordingly, this paper develops a bi-objective integer programming model to address the flight departure scheduling of the partly-restricted (e.g., lower-priority) one among several adjacent airports. An adapted tabu search algorithm is designed to solve the current problem. It is demonstrated from the case study of Tianjin Binhai International Airport in China that the proposed method can obviously improve the operation efficiency, while still realizing superior equity and regularity among restricted flows.

## Introduction

During the past decade, tremendous growth in air traffic demand has been made due to the rapid development of economics worldwide. For instance, as an emerging-market economy, the air traffic demand of China has grown steadily since 2006 and reached 7.5 million by 2014, enjoying an average annual growth rate of 10.8% [1]. The tremendous growth breaks the balance between the demand and supply capacity, and thus leads to airspace congestion and flight delay, which challenge the conventional Air Traffic Management (ATM) approaches [2]. Considering that it is usually practically infeasible (or economically unacceptable) to launch new facility constructions, an effective alternative is to mine the potential of the existing facilities by developing more scientific managements. To this end, arrival scheduling and departure scheduling provide two powerful tools. Both aim to make full use of the runway: the former achieves it by optimizing the landing time of flights, while the latter realises it by optimizing the take-off time. In modeling, the difference between two scheduling problems spans the separation rules (e.g., only successive separations requirements which are imposed between consecutive operations at the runway limits the former), constraints, and objective functions [3]. Studies regarding these two problems are fruitful, especially the former (e.g., [4–15]). Considering that the airport (i.e., Tianjin Binhai International Airport(TJN)) addressed in this paper possesses two runways and each one of them almost just executes a single task (e.g., landing or take-off) during a day, this paper just limits the scope onto the latter. Accordingly, the subsequent literature review is only regarding closely-relevant studies in the departure scheduling.

Ernst et al. [16] suggested a network simplex algorithm to solve the departure scheduling problem (DSP) of flights. Bolender [17] formally incorporated the merging of departure routes into DSP when the airspace was congested. Leeuwen et al. [18] introduced the constraint satisfaction techniques into a relatively small-scale DSP. Gotteland and Durand [19] proposed a combined model which integrated both departure and ground taxi-out, whereas only the wake separation rules was considered to simplify the problem. Atkin et al. [3, 20, 21] elaborated on the DSP of London Heathrow Airport with a special holding point structure, where the possible constrains associated with runway control were considered, and, what is more, the separation was variable with time. It was commonly strengthened that the separation requirements would become complicated if a triangle inequality did not hold. Balakrishnan and Chandran [22] showed a class of techniques based on dynamic programming under CPS constraints that can determine efficient departure schedules that satisfy the various upstream and downstream constraints imposed on the departure runway system. Rathinam et al. [23] addressed a departure scheduling problem with an objective to reduce total aircraft delays subject to timing and ordering constraints. A generalized dynamic programming approach is presented to solve the departure scheduling problem optimally. Gupta et al. [24] proposed a multi-objective Mixed Integer Linear Program (MILP) model for DSP. Seeing the possible high delay resulted from Gupta’s model, Su et al. [25] latter presented a modified MILP model, where a saturation term was added into the objective function to penalize the high delay. Recently, Yin et al. [26] took the pollutant emission into account and developed a multi-objective optimization model to deal with the multi-runway DSP with independent departure mode. In addition, an elitist non-dominated sorting genetic algorithm was adopted to identify the Pareto solutions.

Note that the above-reviewed studies commonly deal with an independent airport in which the departure traffic are not affected by surrounded airports, which, however, is not consistent the case. In reality, there still exist some cases where several commercial airports are closely located and one of them possesses a higher priority, e.g., the Beijing Capital International Airport (PEK) and the Tianjing Binhai International Airport (TJN) in China. In this paper, we refer to a higher priority of an airport like this, during the peak hours, the departure activities of the lower-priority airports are usually required to give way to those of higher-priority ones. The influence resulted from these giving-way requirements can be significant, and thus inflict a variety of changes on the problem modeling of departure scheduling to the lower-priority airports. To the best of our knowledge, studies on the DSP under this condition is scarce. Built on the existing studies, this paper develops a multi-objective model to address the flight departure scheduling of the partly-restricted (e.g., lower-priority) one among several adjacent airports. For better reflecting the reality, time-dependent separations and the interaction between traffic direction flows are considered. An adapted tabu search algorithm is designed to solve the problem. It is demonstrated in the numerical case study that the proposed method can improve efficiency, while still realizing equity among restricted flows.

The remainder of this paper is organized as follows. Section of Problem description conducts a comprehensive description on the partly-restricted DSP discussed in this paper. Section of Formulation then elaborates on the mathematical modeling. In Section of Algorithm, a tabu search algorithm tailored to this problem is designed. A numerical case study for the TJN is implemented in Section of Case study to examine the proposed model and algorithm. Section of Conclusion concludes the whole study and suggests some future works.

## Problem description

**Fig 1:**
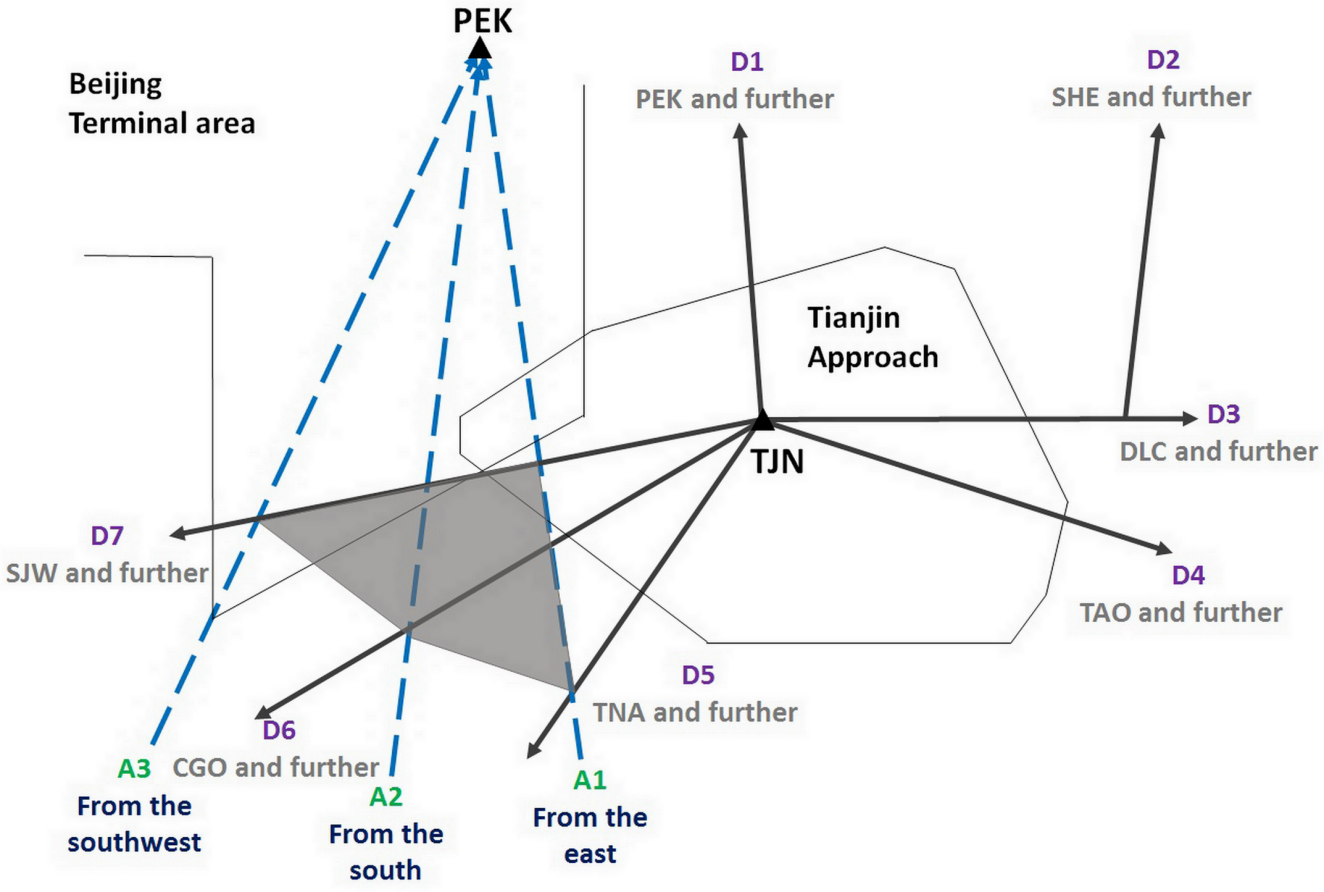
Air traffic direction flow indication of Tianjin. The blue dotted and the black solid arrows indicate the arrival traffic flows to PEK and the departure traffic flows from TJN, respectively. The shadow depicts the overlapped area between two airports.

As mentioned in previous, the difference in departure priority can yield significant negative influence onto the lower-priority airport, which makes the DSP of a lower-priority airport distinguished from that of the independent airport. The departure management normally relies on the Standard Instrument Departure (SID) of flights. Each SID allocates a path to a specific flight and tells this flight a determined departure route. When the departure traffic of an airport is partly restricted, the departure traffic management will change accordingly and the existing SID-based departure management is not applicable any more. To illustrate the problem, as an example, PEK and TJN in China are employed. Fig 1 displays a distribution sketch of selective air traffic directions with respect to PEK and TJN.

In Fig 1, there are 7 major departure traffic flows from TJN. The origions/destinations of air traffic in PEK and TJN are mainly located in south China. For TJN, directions D5, D6 and D7 seize approximately 70% of the whole departure traffic. For PEK, directions A1, A2 and A3 seize nearly 75% of the whole arrival traffic. Statistics show that Beijing airport peak daily traffic volume is up to 1,800 movements, while this figure in Tianjin airport is only 450 [1]. The difference between the traffic volume and the city administrative level determines that the PEK has a higher priority. As can be seen the shadow area in Fig 1, TJN’s southbound departure traffic is influenced by PEK’s arrival traffic from the south, which result in the former receives more ground delay during the arrival peak hours of the latter. In order to accelerate the traffic flow and reduce workload, the capping technique [27] is adopted by very few flights of TJN to avoid excessive delays, which means departure flight has a very low level to crossover the arrival flow of PEK from below. Of course, this fall short of satisfy airlines and passengers in terms of fuel consumption and service quality. Meanwhile, if controller workload reaches the upper bound as a result of continuous traffic accumulation, the air traffic flow managers in PEK will combine certain direction flows of TJN for a period of time. For instance, a common accepting rate for D5, D6 and D7 is adopted during this so called restricted time period. This method is similar with sector combination or splitting in approach control or enroute control.

**Fig 2:**
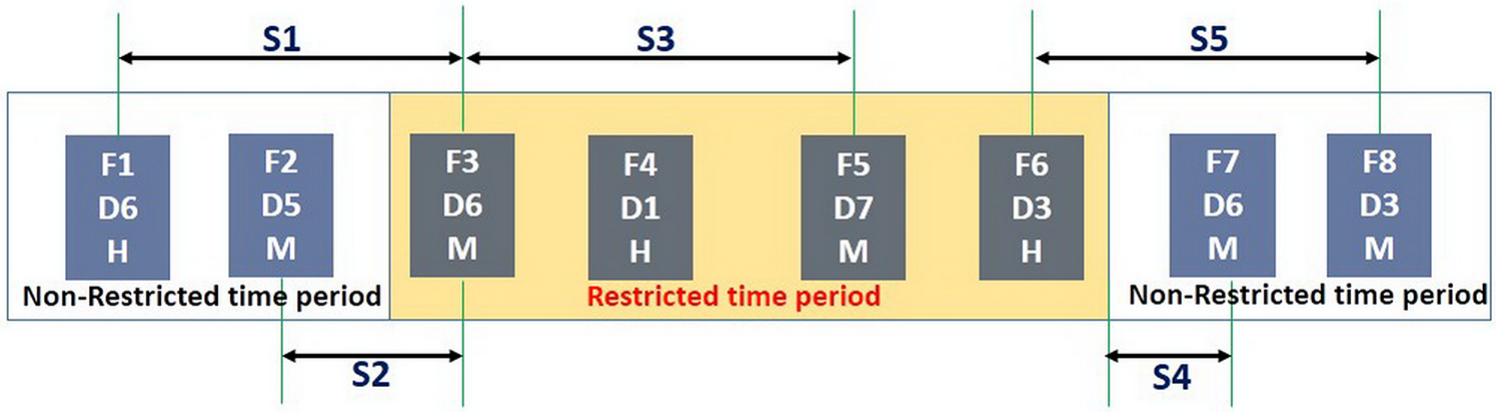
A sketch of separations related to restricted time periods for departure flight sequences. Each blue box gives the related information of a fight; take the first one for example, F1 is the flight sequence, D6 is departure direction of F1, and H is the heavy wake turbulence category of F1. S1-S5 denotes different types of separations.

For a normal DSP, two kinds of separation constraints (i.e., the successive and complete ones [8]) are usually considered. The successive constraints are associated with two consecutive aircrafts at the runway (e.g., wake vortex separation standards), while the complete ones are made for the two non-consecutive aircrafts assigned to either the same departure fix or identical destination (e.g., Minutes-In-Trail separation). Besides to those normal separations, the addition of restricted time periods inflict new changes on the separations. Fig 2 gives a sketch with respect to these changes, still taking the TJN in Fig 1 as an example.

In Fig 2, S1 and S5 are the Minutes-In-Trail separations that need to be maintained for both non-restricted and restricted time periods. Due to the addition of restricted time periods in peak hours, the initially independent Minutes-In-Trail separations will become related. This change leads to the separations shown in S2, S3 and S4, which can be categorized as the following two types.

1. Time-dependent separation on restricted flows
  This is separation only works in a specific time period. In the restricted time period, the accept rates for D5, D6 and D7 are combined as one. Since F3 is within that period, S2 represents the specific Minutes-In-Trail separation between F2 and F3. If F3 is not within that period, S2 will represent wake turbulence separation between them. S3 is in the same situation.
2. Boundary interval
  It represents the interval between the finish boundary of the restricted time period and the calculated take off time of F7, such as S4. In this case, if the calculated take off time of F7 right on the boundary of restricted time period, it will take specific direction flow separation into account with F5, but 1 minute later, there will not be such a requirement.

The change of accepting rate for restricted direction flows results in larger separations in restricted time periods, which eventually leads to increasing flight delay and naturally worsens the flight punctuality. The mitigation measures for this adverse effect is worth exploring.

According to the statistical standard of civil aviation for flight punctuality in China, the ATA and ATD in statistical standard for flight punctuality are based on aircraft actually landing on the runway and starting the takeoff roll respectively. There are two ways for an airline to achieve flight punctuality. One way is starting takeoff roll on the runway no later than a specific time limit after ETD as presented in Table 1. Another way is landing on runway no later than 10 minutes after ETA. The flight is regarded as punctual if either requirement is met.

**Table 1:** Specific time limit for flight punctuality.

Airport classification	Time limitation(min)
PEK, PVG, CAN	30
SHA, SZX	25
CTU, KMG, TJN	20
The rest airports	15

**Fig 3:**
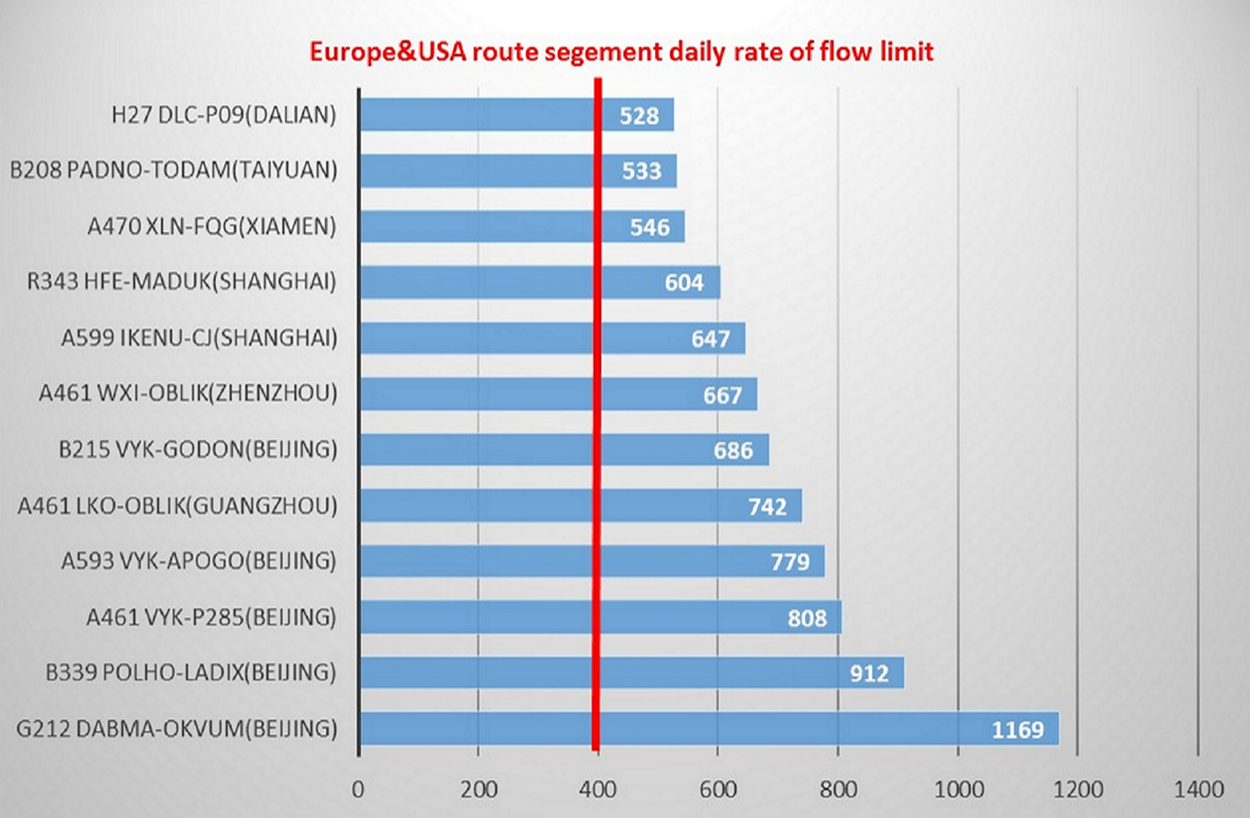
Busy route segment daily rate of flow of China in 2014 [1].

In actual operation, the daily flow rate for the route segments are extremely high in China which are presented in Fig 3. However, this rate in Europe and America is limited to 400. If traffic surpasses the threshold value, the responsible air navigation service provider will activate alternate parallel routes or divert air traffic [1]. The reason for this situation in China is that only 20% of the airspace is available for civil aviation [28]. This extremely congested route network brings huge uncertainty to arrival management. Particularly the congestion often appears in the terminal areas, which makes air carrier always focus on the earliest takeoff time due to flight punctuality consideration. It means even though there are two ways to achieve flight punctuality, which are departure on schedule and arrival on schedule. The former is more preferred than the latter.

Through the above analysis, a model has been developed in order to deal with changes in the operating factors caused by restricted time period. This will be covered in detail in Section of Formulation.

## Formulation

In this section, a mathematical model is developed to deal with the DSP with newly changes illustrated in Section of Problem description.

### Notations and Symbols

For facilitating the statement, as well as helping readers to understand the context better, the notations used throughout this paper are listed as follows in prior.

Sets and indices
*I*(*i*, *j* ∈ *I*): set of total departure flights
*I*^*s*^: set of starting departure flights
$I_{dl}^{s}$: set of delayed starting departure flights, $I_{dl}^{s}\subset I^{s}$
*I*^*c*^: set of connection departure flights
*T*: time horizon of schedule
*T*_*R*_: restricted time period, *T*_*R*_ ⊂ *T*
*F*(*f* ∈ *F*): set of departure directions
*F*_*R*_: set of departure directions affected by restricted time period, *F*_*R*_ ⊂ *F*
Parameters
$T_{R}^{b}$: the start time of restricted time period
$T_{R}^{e}$: the finish time of restricted time period
*p*_*i*_: position of flight *i* in the departure sequence
${\bar{p}}_{i}$: position of flight *i* in the original scheduled departure sequence
*ω*: wake turbulence separation
*ETD*_*i*_: estimated time of departure of flight *i*
*RETD*_*i*_: revised estimated time of departure of flight *i*
*ETA*_*i*_: estimated landing time of the preceding flight of flight *i*
*ATA*_*i*_: actual landing time of the preceding flight of flight *i*
*δ*_*i*_: starting delay of flight *i*
*τ*_*f*_: required time separation imposed on two consecutive flights that join the same departure flow *f*
*τ*^*R*^: required time separation imposed on two consecutive flights that join departure flow which effected by restricted time period
$T_{i}^{out}$: the mean time between flight *i* leaving its gate or parking stand and takeoff
$T_{i}^{in}$: the mean time between flight *i* landing on the runway and returning to its gate or parking stand
$T_{i}^{TA}$: turnaround duration time of flight *i*
$T_{i}^{min-TA}$: required minimum turnaround duration time of flight *i*
$T_{i}^{gs}$: ground service time of flight *i*
*γ*: flight punctuality evaluation criteria, the detailed information is presented in Table 1, *γ* for TJN is 20mins
*α*_*f*_: weight of departure flow *f*
*ε*_*i*_: *ε*_*i*_ = 1 if flight *i* is a delayed starting flight, and *ε*_*i*_ = 0 otherwise
$\sigma_{i}^{f}$: $\sigma_{i}^{f}=1$ if direction flow *f* is the departure direction flow of flight *i*, and $\sigma_{i}^{f}=0$ otherwise
*π*: the pre-setting flight punctuality value
Functions and intermediate variables
*φ*_*i*_: indicator of the regularity state of flight *i*. if the difference between assigned CTOT and ETD of flight *i* is no more than *γ*, *φ*_*i*_ = 1; Otherwise, *φ*_*i*_ = 0
*ξ*_*i*_: 0-1 variable, if flight *i* takes off within the restricted time period, *ξ*_*i*_ = 1; Otherwise, *ξ*_*i*_ = 0
*D*(*x*): the average departure delay during the time period within restricted time periods
*D*_*f*_(*x*): the average departure delay of a specific departure flow *f* during the time period within restricted time periods
Decision variable
*x*_*i*_: the calculated take off time of flight *i*

### Model

The objectives of the current problem are stated as follows.

$$\begin{array}{c} O_{1}=\min\sum_{i\in I}\left(x_{i}-ETD_{i}\right) \end{array}$$ (1)

$$\begin{array}{c} O_{2}=\min\sum_{f\in F}\alpha_{f}\cdot {\left[D\left(x\right)-D_{f}\left(x\right)\right]}^{2} \end{array}$$ (2)

where

$$D\left(x\right)=\frac{\sum_{i\in I}\xi_{i}\cdot \left(x_{i}-ETD_{i}\right)}{\sum_{i\in I}\xi_{i}}$$

and

$$D_{f}\left(x\right)=\frac{\sum_{i\in I}\xi_{i}\cdot \sigma_{i}^{f}\cdot \left(x_{i}-ETD_{i}\right)}{\sum_{i\in I}\xi_{i}\cdot \sigma_{i}^{f}},\;\forall f\in F,\;x_{i}\in T_{R}$$

Objective *O*_1_ focuses on efficiency which aims to reduce the total departure flight delay for the entire time span. However, during the restricted time periods, objective *O*_1_ may lead to huge delay in certain departure flows due to their combination and sharing a common accepting rate. It is indispensable to maintain a reasonable equity level among them. Thus, objective *O*_2_ is designed to minimize the delay differences among diverse directions for restricted time periods.

The constraints of the problem are as follows.

$$\begin{array}{c} x_{i}\geq RETD_{i},\;\forall i\in I \end{array}$$ (3)

$$\begin{array}{c} x_{i}-RETD_{i}\leq 2\gamma ,\;\forall i\in I \end{array}$$ (4)

$$\begin{array}{c} p_{i}=1,\;i\in I\Rightarrow x_{i}=RETD_{i} \end{array}$$ (5)

$$\begin{array}{c} |p_{i}-{\bar{p}}_{i}|\leq k,\;\forall i\in I \end{array}$$ (6)

$$\begin{array}{c} \sigma_{i}^{f}\neq \sigma_{j}^{f}\;\forall f\in F\Rightarrow x_{j}-x_{i}\geq \omega_{ij},\;\forall i<j,\;i,j\in I \end{array}$$ (7)

$$\begin{array}{c} \sigma_{i}^{f}+\sigma_{j}^{f}=2\Rightarrow x_{j}-x_{i}\geq \tau_{f},\;\forall f\in F,\;i<j,\;i,j\in I \end{array}$$ (8)

$$\begin{array}{c} \sum_{f\in F_{R}}\sigma_{i}^{f}+\sum_{l\in F_{R}}\sigma_{j}^{l}=2\;\text{and}\;x_{i}\notin T_{R},\;x_{j}\in T_{R}\Rightarrow x_{j}-x_{i}\geq \tau^{R},\;\forall i<j,\;i,j\in I \end{array}$$ (9)

$$\begin{array}{c} \sum_{f\in F_{R}}\sigma_{i}^{f}+\sum_{l\in F_{R}}\sigma_{j}^{l}=2\;\text{and}\;x_{i},x_{j}\in T_{R}\Rightarrow x_{j}-x_{i}\geq \tau^{R},\;\forall i<j,\;i,j\in I \end{array}$$ (10)

$$\begin{array}{c} \sigma_{i}^{f}\neq \sigma_{j}^{f}\;\forall f\in F_{R}\;\text{and}\;x_{i}\in T_{R},\;x_{j}\notin T_{R}\Rightarrow x_{j}-x_{i}\geq T_{R}^{e}+1,\;\forall i<j,\;i,j\in I \end{array}$$ (11)

$$\begin{array}{c} \frac{\sum_{i\in I}\varphi_{i}}{\left|I\right|}\;\geq \pi \end{array}$$ (12)

Constraint (3) ensures that no flight should take off before it is ready. Constraint (4) prevents the aircraft from being moved further in the schedule and received a patch of huge delay, the latest acceptable CTOT is limited within 2 times of predefined delay statistical standard. The *γ* value of our target airport is 20 minutes. The first flight is assumed to take off at its requested take-off time by Eq (5).

Employing a pre-setting number *k* represents the Maximum Position Shifting (MPS), Constraint (6) ensures some degree of fairness by restricting the final schedule to deviate significantly from the FCFS schedule.

Constraints (7) and (8) ensure flights pair satisfy the wake turbulence separation minima and Minutes-In-Trail separation which form different and same departure flows respectively.

To deal with the accepting rate combination of direction flows analyzed in Section of Problem description, besides Constraints (3) to (8), two additional constraints are needed. Constraints (9) and (10) ensure the flights from restricted departure flows meet pre-setting takeoff separation requirement between the later and former aircraft in the departure sequence during restricted time period.

When the restricted time period is finished, flight *j* is the first flight after it. Constraint (11) ensures flight *j* has opportunity to use the first time after the end of the restricted time period.

Since flight punctuality is a performance evaluation index for air traffic management, it is treated as Constraint (12) which aims to ensure the proportion of non-delayed flights no less than a pre-setting value.

**Note**. Minimizing Eq (1) under constraints Eqs (3)–(8) lead to the model of a normal DSP (e.g., Balakrishnan and Chandran [22]; Rathinam et al. [23]; Gupta et al. [24]), implying that equity (described by Objective Eq (2)) is beyond the consideration of normal DSP, and such a partly restricted condition mentioned in this paper (captured by constraints Eqs (9)–(12)) is not addressed in normal DSP, either.

## Algorithm

Beside MIP, dynamic programming (DP) serves as another alternative to formulate air traffic scheduling problem, e.g., ASP (e.g., Lieder et al. [14]) and DSP (e.g., Balakrishnan and Chandran [22], Rathinam et al. [23]). To make DP adapt to either ASP or DSP, a necessary condition is the satisfaction of a triangle inequality regarding separations for each aircraft. For ASP, such a triangle inequality holds readily since a landing aircraft is only restricted by the wake turbulence separation of consecutive aircraft. In Balakrishnan and Chandran [22], and Rathinam et al. [23], this triangle inequality was assumed to be satisfied. Actually, for DSP, such a triangle inequality does not hold since, beside the wake turbulence separation, a departing aircraft is additionally restricted by the Minutes-In-Trail separation which depends on all previous departed aircrafts. Therefore, we treat it as a MIP problem and apply Tabu Search (TS) to search for the optimal take-off sequences. TS is a metaheuristic approach designed to find a satisfactory solution of combinatorial optimization problem which is first introduced in Glover [29]. Since then it has been applied to various problems. Different from heuristics which are known to be too problem dependant, metaheuristics are more generalized approaches able to solve larger problems better and faster [30]. Previous work Atkin et al. [20] shows that it is an efficient algorithm to solve the departure scheduling problem by comparing with other local search algorithms, such as first descent, steeper descent and Simulated Annealing.

There are five key elements of TS:

1. Initial solution
  The tabu search always starts from the last suggested take-off sequence, or the First-Come-First-Served (FCFS) order if there is no previous suggestion.
2. Neighborhood searching
  Neighborhood *N*(*S*) is defined as a constrained 2-opt. A neighbor is generated by swapping the positions of two aircraft in *S* while complying with the MPS. A candidate set *C*(*S*) is used to mitigate the computational burden. If *n*(*N*(*S*)) > 100, randomly generate 100 new neighbors to make up *C*(*S*); else select the whole neighborhood *N*(*S*) as candidate set *C*(*S*).
3. Tabu list
  A tabu list is built up from the history of moves used to explore search space until the old solution area is left behind. Whenever a move is made, details of the aircraft which were moved and where they were moved from are added as a single entry to a tabu list. The last 20 moves of each aircraft are recorded in it.
  The aspiration criteria will be used in the process of iteration. It is that when all solutions in candidate set are forbidden, then the smallest one objective value of the top 10 non tabu objects as the current solution.
4. Measuring Function
  A new starting point will be selected from candidate set through the measuring function. Eqs (9) and (10) are adopted as the measuring function.
5. Stop Condition
  The algorithm stops after it has run for a predetermined number of iterative steps *MAX*_*iter*_. Another condition that algorithm terminated is when the both objective values of *O*_1_ and *O*_2_ do not decrease in limited steps *MAX*_*opt*_. *MAX*_*iter*_ = 1000 and *MAX*_*opt*_ = 200 are adopted for the numerical experiment.

According to the model and the problems in the Section of Problem description, the following algorithm procedure is designed.

**Algorithm 1**.

**Initialization**

Get an initial solution *X*_*now*_ as described in Section of Input data and parameters. Let the iteration number runcount = 0, the *run*_*opt*_1 and *run*_*opt*_2 represent the number of optimal solution occurs for *O*_1_ and *O*_2_ respectively, the current optimal objective value of *O*_1_ and *O*_2_ are denote as *o*^td^*, *o*^ad^* respectively, and set tabu list *L* = ∅.

**for** *MAX*_*iter*_ iterations **do**

 **if** runcount<*MAX*_*iter*_ and *run*_*opt*_1, *run*_*opt*_2<*MAX*_*opt*_ **then**

  generate 100 random neighbouring solutions from the current solution

  for each of the newly generated solutions

   **if** the solution is tabu **then**

    the smallest non-tabu object is selected as the current objective value

    denote as $o_{now}^{td^{\prime }}$ and $o_{now}^{ad^{\prime }}$ respectively

    $o^{td*}=o_{now}^{td^{\prime }}$, *run*_*opt*_1 = *run*_*opt*_1 + 1

$o^{ad*}=o_{now}^{ad^{\prime }}$, *run*_*opt*_2 = *run*_*opt*_2 + 1

   **else**

    evaluate the solution

    $o^{td*}=o_{now}^{td}$, *run*_*opt*_1 = 1

    $o^{ad*}=o_{now}^{ad}$, *run*_*opt*_2 = 1

   **end if**

  runcount = runcount + 1

  select *o*^td^* and *o*^ad^* as the new current solution

  add details of the move into tabu list *L*

 **else break**

**end for**

## Case study

In this section, we apply our model and algorithm to an actual operation scenario in order to explore the potential benefits. It is necessary to note that PEK is surrounded (and thus partly interfered) by three airports, i.e., TJN, SJW and NAY. During the peak hours of PEK, these three airports are restricted in varying degrees. In accordance with Section of Problem description and without loss of generality, this section still chooses TJN as the low-priority counterpart of high-priority PEK to perform the case study.

### Input data and parameters

This paper adopts a sequence in order of ascending Revised Estimate Time of Departure (RETD) which includes starting and inbound delay as initial solution. The motivation is to fully capture the impact of pre-departure delays. Because China’s aviation market is in a period of rapid growth, airline’s transport capacity shortage and airspace congestion coexistence. The main reasons for flight delay in China in 2014 are airline itself, air traffic flow control and weather impact, accounting for 26.41%, 25.33% and 24.34% [1]. In addition, aircraft mechanical failures and inbound delay are the main reasons of airline delay. In spite of recovery measures, very limited actions can be taken in the event of a shortage of airlines transport capacity. Therefore, airline itself has a significant impact on flight delay in China which can not be ignored. The procedure for generating RETD is described as follow.

The flight data of TJN on 15th October, 2015, collected from Air Traffic Management Sub-bureau of Tianjin is adopted. There are a total of 195 flights that are scheduled to depart from TJN during that day. The flights from 07:00 to 21:00 are selected, altogether 169 flights as the input in experiment. There are two kinds of departure in our schedule, one is the starting departure flight which conducts the first flight segment on the flight cycle and no preceding flight before. The other is the connection departure flight which situates in the middle of flight segment rotation, the inbound delay of preceding flight may influence the departure of succeeding flight.

For starting departure flights, if they miss their ETD, air traffic control will record their actual reported ready time and note ‘late contact’ for them. The difference between ‘late contact’ time and ETD of flight *i* is the starting delay. If there is no starting delay for starting departure flight *i*, it will depart according to their original schedule time, thus

$$\begin{array}{c} RETD_{i}=ETD_{i}+\varepsilon \cdot \delta_{i},\;\forall i\in I^{s} \end{array}$$ (13)

$$\begin{array}{c} \varepsilon =\{\begin{array}{ccc} 1, & & i\in I_{dl}^{s}\\ 0, & & otherwise \end{array} \end{array}$$

For those connection departures, the different inbound delay of preceding flights will result in ground service duration change, thus

$$\begin{array}{c} RETD_{i}=ATA_{i}+T_{i}^{in}+T_{i}^{TA}+T_{i}^{out},\;\forall i\in I^{c} \end{array}$$ (14)

$$\begin{array}{c} T_{i}^{TA}=\max\left(T_{i}^{min-TA},\;T_{i}^{gs}\right) \end{array}$$ (15)

Fricke and Schultz [31] use 24,740 flight operations of A320 data from DLH to explore the relationship between inbound delay and turnaround duration time. The statistical results indicate that the mean value of turnaround time $t_{i}^{gs}$ decreases with inbound delay increases. The detail results are introduced as follow:

$$\begin{array}{c} T_{i}^{gs}=\{\begin{array}{ccc} 58 & & if\;ATA_{i}-ETA_{i}<5\\ 54 & & if\;5\leq ATA_{i}-ETA_{i}<10\\ 51 & & if\;10\leq ATA_{i}-ETA_{i}<15\\ 50 & & if\;15\leq ATA_{i}-ETA_{i}<20\\ 51 & & if\;20\leq ATA_{i}-ETA_{i}<25\\ 49 & & if\;ATA_{i}-ETA_{i}\geq 25 \end{array} \end{array}$$

$T_{i}^{min-TA}$ depends on the number of seats of flight *i*. The detail requirement is presented in Table 2.

**Table 2:** The minimum turnaround time requirement.

Seats of aircraft (SN)	minimum turnaround time requirement (min)
SN ≤ 60	35
61 ≤ SN ≤ 150	50
151 ≤ SN ≤ 250	60
SN ≥ 251	75

The average taxi out time of Tianjin airport is 19.1 minutes [1]. In our experiment, we assume that taxi out time equals to taxi in time. Thus, the revised estimate time of departure is generated which can represent the departure demand shifting.

Table 3 shows the statistical data of departure flights for each departure flow during the period.

**Table 3:** Number of departure flights from 07:00 to 21:00.

Departure flow	D1	D2	D3	D4	D5	D6	D7	total
Number of departure flights	10	14	28	8	42	38	29	169

According to Civil Aviation Air Traffic Management Rules of China, the minimum time separation between two consecutive departure flights shall comply with wake turbulence separation minima. The detail requirement is shown in Table 4.

**Table 4:** Wake turbulence separation minima for consecutive departures (min).

**Leading type**	**Trailing type**			
	super-heavy	heavy	medium	Light
super-heavy	2	2	3	4
heavy	2	2	2	3
medium	2	2	2	2
light	2	2	2	2

The operation log records 3 restricted time periods which are 08:00-11:40, 13:30-14:30 and 16:00-17:30 on that day. During those restricted time periods, D5, D6 and D7 will be combined and share a common accepting rate for Beijing area control center. The detailed required time separations for different departure flows according to flight destination are listed in Table 5.

**Table 5:** Minutes-In-Trail requirement at departure flow (min).

Departure flow	Time period	D1	D2	D3	D4	D5	D6	D7
Minutes-In-Trail separation	N-RTP	WT	WT	4	WT	8	8	8
	RTP	WT	WT	4	WT	8		

**Fig 4:**
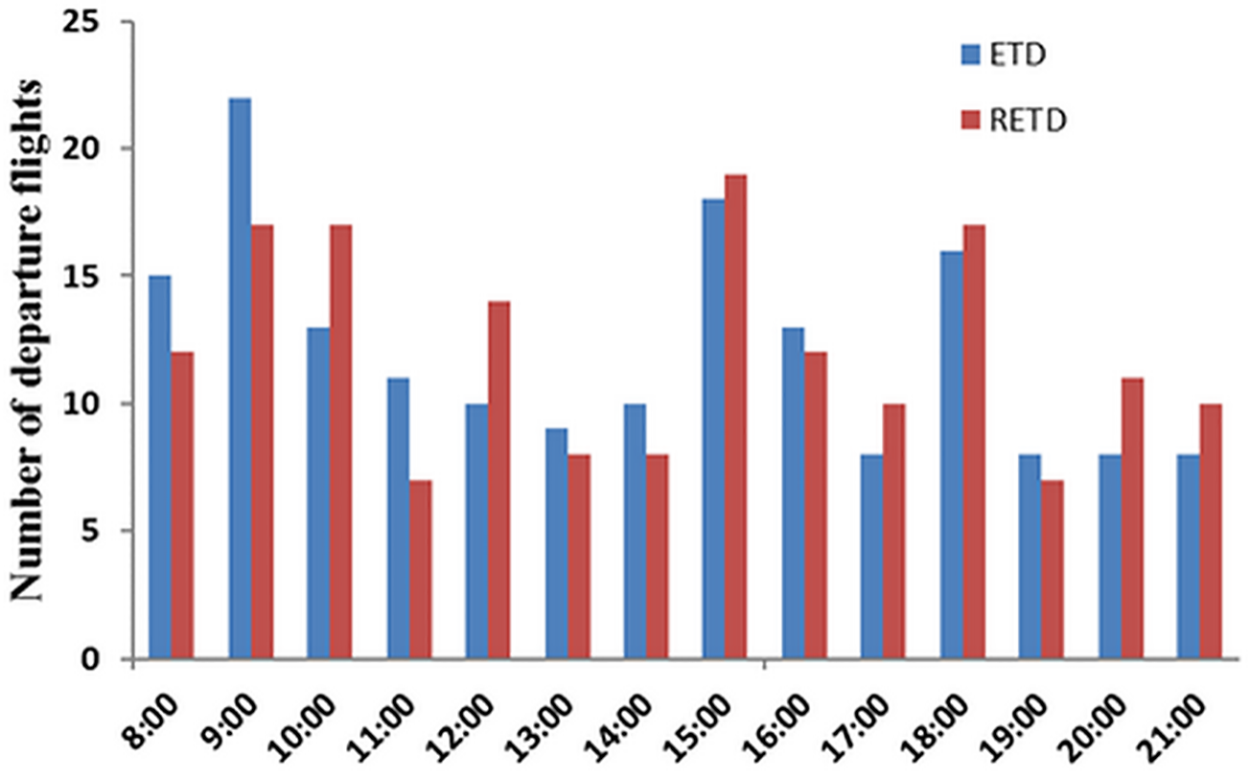
Departure demand distribution for each hour at Tianjin airport on Oct.15, 2015.

The predetermined number of MPS is denoted as *k*. According to Constraints (1) and (2), the adjustment range for individual flight delay is between RETD and 2*γ*. *γ* is determined as 20 minutes from Table 1 which result in the maximum allowable delay is 40 minutes. In contrast to the problems faced by Atkin et al. [3], the departure rate of Tianjin Airport is not as high as London Heathrow Airport which means that flights are relatively sparse in the departure sequence of Tianjin Airport. Fig 4 shows that the hourly number of departures are even less than 10 in some time periods. In addition, Minutes-In-Trail separations in busy traffic direction flows are relatively large. Therefore, moves the flight far away from its RETD position will exceed the delay adjustment range and *k* is set to 3 in our experiment.

The weight of each direction is determined by traffic volume. A 31-day operation data of October 2015 is adopted. The statistics of the proportion of traffic flow in each direction is presented in Table 6.

**Table 6:** The weight of departure flow.

Departure flow	D1	D2	D3	D4	D5	D6	D7
*α*_*f*_	0.07	0.08	0.13	0.04	0.25	0.25	0.19

### Result analysis

The experiment result on the RETD schedule generating shows that the demand shifting has occurred. Fig 4 presents the detail departure demand distributed for each hour, blue column and red column represent departure demand based on ETD and RETD respectively.

**Fig 5:**
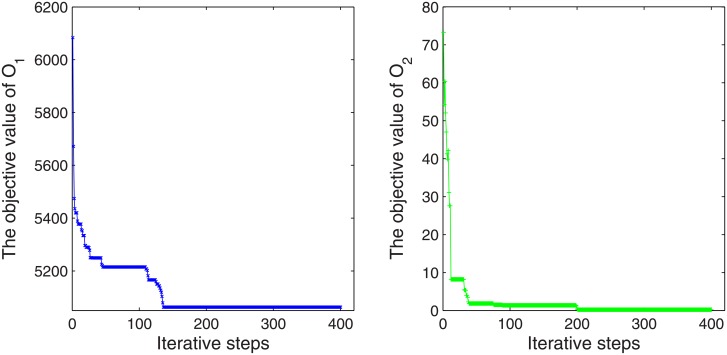
Trends of objectives value in iteration, *π* = 0.44.

Fig 5 displays the trend of individual optimal solutions for the flight delay and the direction average delay difference in restricted time periods corresponding to different iterative steps. As the iterative step continues to increase, the individual feasible solution approaches the optimal direction and searches for the Pareto optimal solution in the decision space. The two objective values from the initial 6084 minutes and 73.28 minutes reduce to 5039 minutes and 1.11 minutes respectively under the condition of flight punctuality is not less than 44%. The overall flight delay is 5039 minutes reveals that the preflight delays have a great impact on the result of inefficient slot utilization. Our selected case data of TJN exist an average of 21.9 minutes preflight delay for each flight. The mitigation practice on the one hand lies in airline to improve transportation capacity and flight supportability. On the other hand, air traffic management unit schedule arrangement should be based on the operation characteristics of close located airports with different priority to collaboratively reduce scheduled delay.

**Fig 6:**
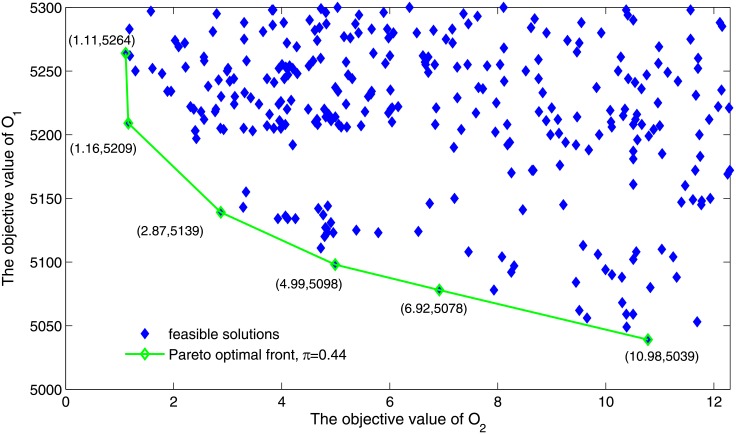
The distribution of solutions at *π* = 0.44.

As observed in Fig 6, the algorithm has searched the Pareto optimal solution when the stop condition is reached. The frontier curve is decreasing, meaning that as delay-saving reaches a higher level, more equity needs to be given up in order to achieve equal improvement in efficiency. Therefore, Air traffic managers can make a comprehensive trade-off between efficiency and equity based on the performance characteristics of the Pareto optimal solution set.

**Fig 7:**
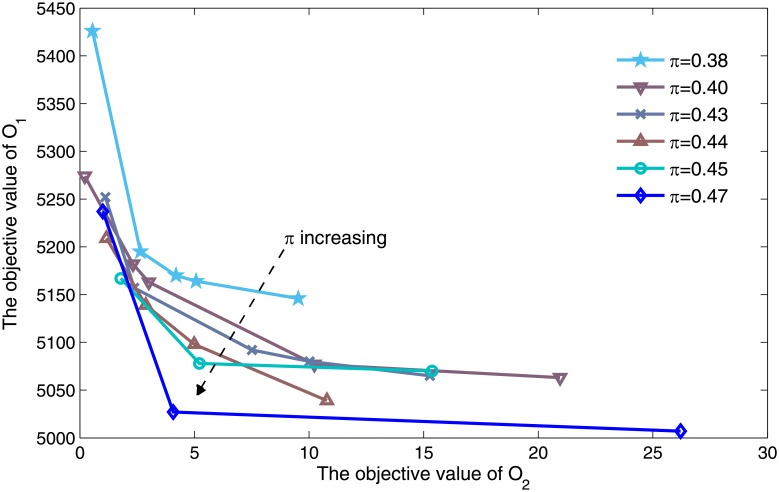
Pareto fronts at different flight punctuality levels.

Flight punctuality *π* as a critical input parameter, its effect on efficiency and equity needs to be further explored. Fig 7 shows the resulting Pareto front under the different flight punctuality constraints. The x-axis and y-axis represent values of overall flight delay and direction average delay difference in restricted time period respectively, while each curve represents a Pareto front under flight punctuality *π* constraints as identified in the legend. The starting value of *π* is set to 0.38 due to flight punctuality of initial solution which based on FCFS is 37.87%. According to the numerical experiment, the trend of Pareto front is divided into two phases. At the first phase, Pareto front moves to the coordinate origin as *π* increasing from 0.37 to 0.47, which means the increase in the flight punctuality improves the Pareto front. Furthermore, an obvious monotonicity is observed, flight delay and regularity show positive correlation, while direction average delay difference appearing negative correlation with them. At the second phase, Pareto front moves away from the coordinate origin when *π* is greater than 0.47. Moreover, no feasible solution is searched when *π* is greater than 0.5. The situation in this phase is triggered by two reasons, first of all, with the increase of *π* is greater than 0.47, the feasible solution space becomes decreasing which limits the range of the Pareto optimal solution. The other reason is that extremely pursuit flight punctuality will lead to the flight has been delayed to be assigned as much as possible delay, which result in overall flight delay and direction average delay difference significantly increase.

In order to demonstrate the potential benefit of the model, different scheduling optimization strategies as well as the actual operation data are employed. There are three Cases that use the single objective to optimize the same RETD schedule, minimize flight delay, minimize the direction average delay difference for restricted time periods and maximize flight punctuality as the only objective for Case A, Case B and Case C respectively. Furthermore, none of them has the flight punctuality constraint. {P1, P2, P3} represent Pareto optimal set, the computational result comparison between them is shown in Table 7. The objective values present different characteristics under different optimization strategies. The minimum flight delay strategy of Case A has the least delay but a lower level of equity. This is due to the fact that flights with larger separations are rescheduled to as late as possible considering the minimizing delay objective function. Applying minimum direction average delay difference strategy of Case B greatly increased the overall delay and decreased the flight punctuality. Because the traffic distribution is not homogeneous, the flights in restricted direction flows have a large proportion in total departure flight and no direction flows combination during non-restricted period, exceedingly sharing delay between direction flows in non-restricted time periods bring greater delay for other direction flows. The maximum flight punctuality strategy of Case C has the maximum flight punctuality, but the remaining objective values are relatively high. This is in accordance with the conclusion discussed previously. In addition, the objective values of *O*_2_ are intensely high for Case A and Case C by comparison with FCFS result, which means applying them separately lead to equity problem in restricted time periods. In contrast, the benefit of our model is that each objective value of Pareto optimal set has been improved but the unfavorable effect is limited.

**Table 7:** The computational results comparison.

	FCFS	Case A	Case B	Case C	*π* = 0.47			Real
					P1	P2	P3	
*O*_1_(min)	6084	4687	5556	5483	5237	5027	5007	6720
*O*_2_(min)	73.28	112.83	0.042	190.52	1.002	4.077	26.21	132.07
*regularity*	37.87%	51.48%	39.64%	56.8%	47.93%	49.70%	50.30%	31.36%

Solutions derived from the proposed model show far less total delay than the real manual results, as well as significantly improved equality among various flow directions. It is still necessary to note that the superior performance of present approach is built on the assumption that the optimized scheme is executed perfectly (similar interpretations were also mentioned in Atkin et al. [20]). As we know, this is challenging in reality due to various interruptions (e.g., meteorological closures, apron condition change). To make more justified comparison with the practically used approach, a direct method is to apply the present approach into practice, and another alternative is to develop a precise simulation for present approach. These works are valuable. However, we will not discuss them here due to the scope of this paper, but leave them to our future works.

## Conclusion

This paper develops a bi-objective integer programming model to address the flight departure scheduling of the partly-restricted one among several adjacent airports, and a tabu search algorithm is designed to solve the model. It is demonstrated from an actual operation case study of TJN in China that i) the current algorithm is effective to solve the proposed model, and that ii) the proposed method can obviously improve the operation efficiency of TJN, while still realizing superior equity and regularity among restricted flows.The presented results show that efficient and equity scheduling flights for takeoff can better utilize restricted resources and reduce the impact of traffic interaction between airports. The current discussion between equity and efficiency can not only deepen our understanding on the interaction mechanism between them, but also provide some theoretical references for practical air traffic managements when equity must be considered.

As for the future works, the primary one can be identifying or developing more efficient algorithms. Based on the current study, integrating the runway configuration change, departure paths and taxiway routing into a united model is also a valuable work.
